# Community social capital on the timing of sexual debut and teen birth in Nicaragua: a multilevel approach

**DOI:** 10.1186/s12889-016-3666-9

**Published:** 2016-09-15

**Authors:** Bomar Mendez Rojas, Idrissa Beogo, Patrick Opiyo Owili, Oluwafunmilade Adesanya, Chuan-Yu Chen

**Affiliations:** 1 International Health Program, National Yang-Ming University, Taipei, Taiwan; 2 École Nationale de Santé Publique, Ouagadougou, Burkina Faso; 3 Institute of Public Health, National Yang-Ming University, Medical Building, Rm 210, 155, Sec. 2, Linong Street, Taipei, 112 Taiwan; 4 Center of Neuropsychiatric Research, National Health Research Institutes, 35 Keyan Road, Zhunan, Miaoli County 350 Taiwan

**Keywords:** Teen birth, Sexual debut, Multilevel, Social capital

## Abstract

**Background:**

Community attributes have been gradually recognized as critical determinants shaping sexual behaviors in young population; nevertheless, most of the published studies were conducted in high income countries. The study aims to examine the association between community social capital with the time to sexual onset and to first birth in Central America.

**Methods:**

Building upon the 2011/12 Demographic and Health Survey conducted in Nicaragua, we identified a sample of 2766 community-dwelling female adolescents aged 15 to 19 years. Multilevel survival analyses were performed to estimate the risks linked with three domains of community social capital (i.e., norms, resource and social network).

**Results:**

Higher prevalence of female sexual debut (norms) and higher proportion of secondary school or higher education (resource) in the community are associated with an earlier age of sexual debut by 47 % (*p* < 0.05) and 16 %, respectively (*p* < 0.001). Living in a community with a high proportion of females having a child increases the hazard of teen birth (*p* < 0.001) and resource is negatively associated with teen childbearing (*p* < 0.05). Residential stability and community religious composition (social network) were not linked with teen-onset sex and birth.

**Conclusions:**

The norm and resource aspects of social capital appeared differentially associated with adolescent sexual and reproductive behaviors. Interventions aiming to tackle unfavorable sexual and reproductive outcomes in young people should be devised and implemented with integration of social process.

## Background

The connection of teen births with an array of adverse outcomes, such as early neonatal death, low birth weight, anemia, postpartum hemorrhage, puerperal endometritis and high caesarean section rate, has been consistently documented [1–4]. In Nicaragua, 20 % of the total population are adolescents [5]. The fertility rate was estimated 89 per 1000 girls aged 15 to 19 years in 2014—the second highest rate in Latin America [5] and teenage girls account for approximately 25 % of births and about half of those births are unintended [6]. The prevalence of sexual debut before 15 years is 13 % [7], consequently heightening the incidence of sexually transmitted diseases and unintended pregnancy [8]. This high fertility occurs in a socio-cultural environment that applauds men with high number of sexual partners and where abortion is illegal [9]. Although adolescent sexual and reproductive behavior are context-sensitive [10, 11], until now most of existing research in this field has mainly provided evidence concerning the effect of individual-level characteristics, as highlighted in recent systematic reviews [12–14].

Findings from prior nationwide representative studies supporting the existence of contextual effect on adolescent reproductive outcomes in Nicaragua are manifested in urban –rural differentials in sexual debut among adolescents [9, 15]. Aside from residence area, smaller geographical units such as neighborhood are of interest for policy decision since they represent a proximate environment where adolescents commonly spend spare time out of their households and create new relationships [16]. Recognizing the neighborhood-based homogeneity in social network, resource, relationship, and norms, an increasing attention has been paid to the potential role and mechanism of neighborhood attributes on teenage reproductive health, especially in high income countries [16]. Among the neighborhood-level factors linked with population health outcomes, social capital is one of the commonly reported neighborhood characteristics [16–20].

The theory based on social capital posits that the interplay of social processes (e.g., social connections and social interaction) will have impact on individual health through the provision of resources and the formation of norms, values, and beliefs [21]. Given its potential malleability, social capital is of relevance to policy makers [22–24]. Nevertheless, the available results pertaining to the connection between social capital and sexual and reproductive health amongst young people were rather mixed, probably due to variation in analytical approach and measure. Several studies in the United States had adopted the ecological study approach [19, 20, 25]. One study reported a negative correlation between social capital (e.g., measures of organizational life, involvement in public affairs and social trust) and teen pregnancy [19], and some suggested that social capital (measured by group membership and social trust) may serve as a mediator for income inequality on teen births [20] and the link between social capital and teen births may be moderated by neighborhood’s ethnic composition when social capital is measured through collective efficacy (a composite scale based on social cohesion and social control) [25]. A noteworthy point is that studies of ecological design are often limited in disentangling the contextual effect from compositional effect [26]. To address this limitation, some scholars have turned to the multilevel approach. A study from the U.K. found that social capital (defined as social cohesion and social control) at neighborhood level is negatively associated with teen birth [17], whereas a study from U.S. reports an insignificant association between neighborhood social capital (defined as social cohesion/trust and social control) and ever having sexual intercourse [18].

In order to address the abovementioned gaps in the literature, the current study used the latest Demographic and Health Surveys (DHS) to examine whether social capital is associated with sexual onset and teen birth in Nicaragua. In this paper we adopted the multilevel approach to tackle potential effects of three perspectives of community-level social capital: social network, social norms, and resource [27]. Social network, indexed by residential stability and religious affiliation [28, 29], reflects the attachment and social interaction with community as well as the degree of participation in community affairs and membership in community organizations [30]. Social norms, which define the acceptability of behaviors and can be viewed as the basis to build and maintain trust [31, 32], were measured by percentage of childbearing women with sex behaviors or already having a child. For resource accessibility, we used the percentage of residents with higher education to index the access to information and material resources for collective efforts [27, 33].

## Methods

### Study design and data

This research used data from the DHS conducted in Nicaragua (years 2011/2012). The DHS is a nationally representative survey, with cross-sectional design selecting households through a two-stage sampling strategy to interview women aged 15 to 49 years and men aged 15 to 59 years. This series of surveys are carried out every 5 years and aim to generate information about maternal and child health. The information was collected by face-to-face interview, and the response rate of eligible women was 91 % [34]. In order to facilitate responses to sensitive issues and decrease social desirability, the DHS’ data collection procedure strongly recommended the respondents to be interviewed by a same-sex member who was also responsible for filling the information given during the survey [35]. In the present study, the analytic sample includes 2 766 females Nicaraguan who were aged 15 to 19 years at survey time. This sample size exceeds the minimum sample size of 1 365 adolescents required to achieve 65 events (i.e. sexual debut) and provides enough statistical power to detect differences in the distribution of the outcome across main predictors [36]. As a proxy for community we used the census tract (*n* = 356), which usually corresponds to a village in the rural area or to a neighborhood in the urban area [37].

### Study variables

#### Outcome variable

The first outcome variable is the age at sexual onset (in years). For teens with no sexual debut at survey time, the age at interview was used to include them in the analysis (i.e., censor under the terminology of survival analysis). The second outcome variable is the time to first birth, defined as the period between sexual debut and the day, month and year of first live birth. We selected first live birth rather than date of first pregnancy because the latter is not available in the DHS. For the variable concerning sexual debut, the respondents were asked the month and year when it occurred. Date of first live birth was recorded from the birth certificate or vaccination card. For teens with no birth (censor) the date of survey interview was used as the ending time.

### Main predictors

This study based its definition of community social capital on Bordieu and Putnam’s theories. The former incorporates the notion of access and utilization of common resources for mutual benefit [21, 27], and the latter concerns “features of social organization that can improve the efficiency of society by facilitating coordinated actions, such as trust, norms and networks” [38]. Given that the formulation of community social capital depends on the existence of trust to others community members and the strength of connections among them [39], we hypothesized that those social processes are more fragile for people who have lived in the same community for less than 10 years (the same cutoff point of 10 years has been used in prior research using DHS) [37, 40, 41]; they may be less socially integrated which may hinder the access to emotional and material resources that operate in favor of health [28]. Therefore, our first main predictor is the proportion of people who have lived in the community for less than 10 years. This variable was created to take into account the responses from men aged 15 to 59 years and women aged 15 to 49 years: each respondent was asked “*How long they have been living in the community*”, and the responses for those who have lived for less than 10 years were aggregated and the mean proportion by census tract was estimated. The second main predictor is related to religious community composition. Because 50 % of Nicaraguans are affiliated with Catholicism, we focused on this religious denomination [42]. The percentages of Catholics in the community were derived using the responses from selected women and men regardless of their age. A similar procedure was followed to estimate the percentage of community inhabitants with no religious affiliation in each census tract. We anticipated this approach allows us to capture the heterogeneous composition of community social networks (e.g., religious institutions) and therefore obtain more accurate estimations of the extent to which the community-level of trust and participation influence adolescent’s reproductive outcomes. As regards social norms, a teenage girls may be influenced not only by their peers but also by adult women’s reproductive behaviors in their surrounding environment (e.g., women from her families or neighborhood), hence we estimated community social norms indicators (i.e., proportion of childbearing age women with sexual debut and having a child) based on the responses of women between 15 to 49 years in each census tract [43]. Finally, the proportion of community members with secondary school or higher education was also estimated by averaging the responses of women aged 15 to 49 years and men aged 15 to 59 years in each census tract. To facilitate the interpretation, all community-level variables were standardized.

### Covariates

At individual-level, we also took woman’s years of education, women’s household wealth index quintiles, marital status (at survey time), woman’s religion, residence area, female age at sexual debut, and prior history of abortion into account by statistical adjustment [12, 13].

### Statistical analysis

The analyses were broken down into two parts. First, to describe the population characteristics and estimate the median survival time to first birth, we used the complex survey analysis to account for a multi-stage sampling design and correct for unequal probabilities of selection [44]; for community variables, central tendency measures were estimated. Second, we implemented survival regression models to estimate the hazard rate of the timing of sexual debut and first birth. Since adolescents are nested within communities, which leads to a violation of the assumption of independent distribution of error terms [26], the utilization of standard Cox regression would lead to imprecise estimations. To overcome this issue, we employed a two-level survival model, with teenage girls as the first level and communities as the second level. Model 1 displays the unadjusted hazard ratios from community- and individual-level variables. In model 2 each contextual variable was adjusted for individual characteristics, and finally we entered all individual- and community-level variables in model 3. For all models we incorporated probability weights at community level. Descriptive analyses were conducted in Stata 12.0 and regression analysis in R 3.2.0 using frailtypack.

### Ethics

All DHS protocols were approved by the ICF’s Institutional Review Board (IRB) [37]. A parent or guardian must provide consent prior to participation of adolescents [35]. The DHS guarantee respondent’s anonymity.

## Results

This study included a total of 2 766 young females aged below 20 years. Table 1 portrays the baseline characteristics. Among the entire surveyed sample, the mean year of education attainment is 7.75, the majority (54.6 %) has less than 7 years of education and has the lowest median time to first sexual experience. People from the lowest wealth index (21.0 %) and those with no religion (15.4 %) have the shortest (16 years) median time to sexual debut. Regarding the subsample of female youth who ever had sex (*n* = 1 247), the median survival time is prominently longer in the following variables: high education (30 months), being in the richer wealth index (34 months), unmarried (30 months), or residing in urban setting (30 months).

**Table 1:** Descriptive statistics of female participants aged 15 to 19 years

Variables	Female youth *N* = 2 766				Female youth who ever had sex *N* = 1 247			
	Mean (SE)	%wt^a^	Median time to first sexual encounter (years)	Log rank test (*p*-value)	Mean (SE)	%wt^a^	Median time to first birth (months)	Log rank test (*p*-value)
Women’s education (years)	7.75 (0.08)				6.90 (0.11)			
Low (0 to 7 years)		54.6	16	<0.001		57.6	25	0.02
High (8 to 16 years)		45.3	19			42.3	30	
Wealth index quintiles								
Poorest		21.0	16	<0.001		26.1	24	0.001
Poorer		16.6	17			20.6	33	
Middle		26.5	18			24.2	31	
Richer		18.4	17			19.5	34	
Richest		17.3	19			9.60	31	
Marital status								
Unmarried		44.4				36.5	30	0.0001
Married		55.5				63.4	25	
Woman’s religion								
No religion		15.4	16	<0.001		20.5	25	0.50
Catholics		44.7	18			40.7	26	
Protestants		37.5	17			37.3	28	
Residence area								
Urban		54.9	18	<0.001		48.9	30	0.002
Rural		45.0	17			51.0	24	
Age of sexual debut (years)								
15 or under						62.5	27	0.80
16–19						37.4	25	
Have you ever had abortions?								
Yes						14.5	27	0.91
No						85.4	27	

Residential stability: stables, the adolescent have lived 10 or more years in the community; unstable, the adolescent have lived less than 10 years in the community

Respondents who belong to other religion were excluded from the analysis

^a^%wt: Weighted percentages

Table 2 shows the distribution of the community variables. The mean percentage of inhabitants that have lived for less than 10 years in the community is 17.7 %, although between-community variation was observed as denoted by the standard deviation (SD) of 13.3 and the interquartile range (IQR) of 9.3 to 23.1. The mean percentage of Catholics at community level is 46.4 % (SD = 23.9, IQR =26.6–64.7), and the average proportion of respondents with no religion in the community is 14.9 % (SD = 10.6, IQR = 7.1, 21.4). The community average proportion of having experienced sexual debut among women with childbearing ages at the time of survey is 85.5 % (SD = 8.05, IQR = 80.6, 91.3), and on average 71.9 % (SD = 11.0, IQR = 64.7, 80) of women aged 15 to 49 years in the community have had at least a child.

**Table 2:** Distribution of community variables (*N* = 356)

Community-level characteristics	Mean	SD	Median	Interquartile range
Percentage of community inhabitants that have lived in the same community 10 or less years	17.7	13.3	14.8	9.3–23.1
Percentage of community inhabitants that are Catholic	46.4	23.9	44.4	26.6–64.7
Percentage of community inhabitants with no religion	14.9	10.6	13.3	7.1–21.4
Percentage of community inhabitants with secondary school or higher	43.1	27.0	40.7	18.5–64.5
Percentage of childbearing aged females with onset sexual debut (age 15 to 49 years)	85.5	8.05	85.7	80.6–91.3
Percentage of childbearing age women currently having a child (age 15 to 49 years)	71.9	11.0	72	64.7–80

Table 3 exhibits the results of three models of survival analysis predicting transition to sexual onset. As indicated in model 1, all community capital variables appeared to be statistically significant except for community inhabitants with no religion. However, with all individual variables adjusted (model 2), only social norms variables remained significant. Finally, model 3, including both community- and individual-level variables, showed that one social norms (i.e., community-onset sexual debut; adjusted Hazard Ratio [aHR] = 1.47, CI 95 % = 1.39–1.56) and social resource (aHR = 1.16, CI 95 % = 1.02–1.33) may increase the girls’ hazard to have sexual onset. As for individual-level variables, the hazard is reduced by 17 % per 1 year of education (95 % CI = 0.81–0.85), 30 % for richest wealth index (*p* < 0.001), and 25 % for Catholics and Protestants (all *p* < 0.05).

**Table 3:** Individual and community characteristics predicting the transition to sexual onset among females aged 15 to 19 years

Variables	Model 1 uHR (95 % CI) *N* = 2 766	Model 2 aHR (95 % CI) *N* = 2 766	Model 3 aHR (95 % CI) *N* = 2 766
Community social capital (z-score)			
% of community inhabitants that have lived in the same neighborhood less than 10 years	1.13 (1.04–1.22)**	1.06 (0.99–1.14)	1.01 (0.94–1.09)
% of community inhabitants that are Catholics	0.88 (0.81–0.95)**	0.92 (0.85–1.00)	0.98 (0.89–1.07)
% of community inhabitants with no religion	1.07 (0.99–1.15)	1.04 (0.97–1.11)	1.02 (0.95–1.10)
% of females in the community with onset sexual debut (aged 15 to 49 years)	1.52 (1.43–1.61)***	1.47 (1.39–1.56)***	1.47 (1.39–1.56)***
% of females in the community aged 15 to 49 years currently having a child	1.33 (1.25–1.43)***	1.14 (1.06–1.24)***	1.07 (0.99–1.16)
% of community inhabitants with secondary school or higher	0.75 (0.70–0.81)***	0.98 (0.85–1.12)	1.16 (1.02–1.33)**
Individual characteristics			
Women’s education (years)	0.81 (0.79–0.82)***		0.83 (0.81–0.85)***
Wealth index quintiles			
Poorest	1		1
Poorer	0.76 (0.63–0.92)***		0.93 (0.76–1.14)
Middle	0.66 (0.52–0.83)***		0.96 (0.74–1.23)
Richer	0.59 (0.49–0.72)***		0.84 (0.66–1.06)
Richest	0.39 (0.31–0.48)***		0.70 (0.54–0.91)***
Woman’s religion			
No religion	1		1
Catholics	0.53 (0.45–0.62)***		0.75 (0.63–0.90)**
Protestants	0.64 (0.54–0.75)***		0.75 (0.62–0.89)**
Residence area			
Urban	1		1
Rural	1.52 (1.33–1.74)***		0.93 (0.71–1.22)

^**^ Significant at 5 % level

^***^ Significant at 1 % level

Model 1.uHR: Unadjusted hazard ratio

Model 2.aHR: Adjusted hazard ratio were estimated entering each community variable separately while adjusting for individual-level variables

Model 3.aHR: Adjusted hazard ratio were estimated with community – and individual-level variables entered simultaneously

All hazard ratios were estimated taking into account household sampling weight

The association estimates for community- and individual- characteristics predicting the transition from sexual debut to the first birth occurrence were summarized in Table 4. With all variables adjusted, we found that a higher percentage of female-onset sexual debut and of residents with higher education may reduce the hazard to have the first birth by 38 % (95 % CI = 0.53–0.70) and 11 % (95 % CI = 0.78–0.99), respectively; whereas a higher percentage of females having a child may elevate the hazard by 76 % (95 % CI = 1.52–2.02). At individual-level variables, women who were married have 36 % (95 % CI = 1.12–1.67) greater hazard of birth occurrence while this hazard was decreased by 5 % per year of education, and was 27 % lower for the poorer wealth index group (95 % CI = 0.54–1.00, *p* = 0.051).

**Table 4:** Individual and community characteristics linking transition from sexual debut to first birth among females aged 15 to 19 years

Variables	Model 1 uHR (95 % CI) *n* = 1 247	Model 2 aHR (95 % CI) *n* = 1 247	Model 3 aHR (95 % CI) *n* = 1 247
Community social capital (z-score)			
% of community inhabitants that have lived in the same neighborhood less than 10 years	1.10 (1.01–1.20)**	1.06 (0.97–1.16)	1.04 (0.94–1.14)
% of community inhabitants that are Catholics	1.00 (0.91–1.10)	0.99 (0.89–1.10)	1.03 (0.91–1.16)
% of community inhabitants with no religion	1.02 (0.93–1.11)	1.02 (0.93–1.12)	1.01 (0.91–1.12)
% of females in the community aged 15 to 49 years with onset sexual debut	0.93 (0.85–1.02)	0.89 (0.81–0.98)**	0.62 (0.53–0.70)***
% of females in the community aged 15 to 49 years currently having a child	1.13 (1.04–1.22)***	1.23 (1.12–1.36)***	1.76 (1.52–2.02)***
% of community inhabitants with secondary school or higher	1.14 (1.06–1.24)***	0.94 (0.83–1.06)	0.89 (0.78–0.99)**
Individual characteristics			
Women’s education (years)	0.94 (0.91–0.96)***		0.95 (0.92–0.99)**
Age of sexual onset			
< 15 years	1		
16 to 19 years	0.92 (0.74–1.15)		1.20 (0.95–1.52)
Wealth index quintiles			
Poorest	1		1
Poorer	0.68 (0.52–0.89)**		0.73 (0.54–1.00)
Middle	0.62 (0.45–0.87)**		0.74 (0.50–1.10)
Richer	0.65 (0.50–0.86)**		0.80 (0.56–1.16)
Richest	0.71 (0.53–0.94)**		1.03 (0.68–1.54)
Woman’s religion			
No religion	1		1
Catholics	0.92 (0.73–1.16)		0.89 (0.68–1.15)
Protestants	0.84 (0.66–1.05)		0.84 (0.65–1.09)
Residence area			
Urban	1		1
Rural	1.46 (1.22–1.74)***		1.11 (0.82–1.49)
Marital status			
Unmarried/no cohabitation	1		1
Married	1.51 (1.25–1.84)***		1.36 (1.12–1.67)**
Have you ever had abortions			
No	1		1
Yes	1.05 (0.82–1.34)		1.05 (0.81–1.35)

^**^ Significant at 5 % level

^***^ Significant at 1 % level

Model 1.uHR: Unadjusted hazard ratio

Model 2.aHR: Adjusted hazard ratio were estimated entering each community variable separately while adjusting for individual-level variables

Model 3.aHR: Adjusted hazard ratio were estimated with community – and individual-level variables entered simultaneously

All hazard ratios were estimated taking into account household sampling weight

## Discussion

On the basis of population-based survey in Central America, our results demonstrated that social capital, manifested in the social norm and resource perspectives, may play an important role in shaping teenage girls’ timing of sexual debut and first birth. The prevailing social norms of female-onset sexual debut and resources may increase the hazard of sexual onset by 47 and 16 %, respectively, yet reduce the hazard of having the first birth by 38 and 11 %.

The lack of significant association for social network suggested that social interactions and social cohesiveness generated within community organizations may have little influence on both sexual debut or early childbearing of very young women [29, 45]. As compared with other religious groups such as Protestants, the Catholicism may be less active in neighborhoods or villages (for example: having fewer churches and offering fewer worship services), which may undermine its influence at the geographical level [46]. Given that religious-affiliated people are known to be more willing to participate in community affairs [47], it is also possible that our measure of religion-related social network did not capture the extent to which community members are actively participating, interacting with each other and eventually creating bonds that allow them to cope with adverse situations [29]. Nevertheless, our study may not be directly comparable with some prior multilevel studies conducted in Kenya, Philippines, and U.S., where different indicators of social network, unit of geographical aggregation (e.g., counties instead of a proxy of neighborhood), and statistical techniques were used [37, 48–50]. Finally, individual-level religion was found to delay the timing of sexual debut, indicating the possibility that religion-related social network (particularly the Catholics) on sexual intercourse may be operated indirectly through individual religious affiliation; for example, this influence could be exerted by inculcating teachings that encourage female adolescents to remain a virgin until marriage [51].

Prevailing social norms at community-level predict sexual onset and transition to the first birth: high community percentage of female- sexual onset exerts an opposite influence on sexual debut and transition to adolescent motherhood. It is possible that the first sexual encounter was a consequence of male counterpart pressure, sporadic, and not part of a plan to build a family [52]. Relationships with those characteristics likely end up in disarray and subsequent abandonment and stigma (for losing virginity) [9] and may make it difficult for girls to build future family or successfully transit to motherhood. Even though no separation occurred after first sexual intercourse, the unmarried girls (as 36.5 % of our analytical sample) may opt for avoiding having a child out of wedlock and being stigmatized [53]. Living in a community with high percentage of females currently having a child predicts faster transition to first birth which may be an expression of social pressure to start motherhood while still young and being able to cope with physical demanding activities associated with motherhood [31, 32].

Our community education variable was shown to influence both sexual onset and first birth. Communities with high percentage of inhabitants with secondary school and higher education are expected to have higher levels of social development [54]. Prior research has established that more developed areas are less likely to follow conservative and traditional lifestyle, thus the observed positive association of community education with sexual onset may reflect departure from traditional norms (e.g., delayed sexual debut) [9, 37]. More developed communities may also offer adolescents more opportunities such as easier access to schooling, employment, or healthcare that may contribute to deter adolescent childbearing [13, 55]. This observation, echoing the individual-level findings that higher education may delay the time to having the first birth, highlighted the importance of education, both individually (a form of human capital) and collectively, in young population in order to have healthy development [56].

This paper has several limitations. First, since the DHS offers a limited set of social capital related-variables, we were unable to use more comprehensive social capital measures used in prior research [19]. Second, although the quality of data collection from DHS is known to be high, social desirability or recall bias may arise when date of sexual debut was collected. Third, due to the cross-sectional design of our study, no temporal sequence can be established. For example, our measurements of community social capital reflect the characteristics of the current place where the respondent live and not necessarily the characteristics before the outcome occurred. Finally, our results from the subsample of adolescents who have ever had sexual encounters should be interpreted with caution since the younger respondents (e.g., 15-years) are more vulnerable to be right censored even if they had the first sexual debut at the same age with those of the 19-year old.

In spite of these limitations, this paper is one of the first studies examining the role of community social capital on adolescent sexual behavior in the context of middle income countries. Further, the sampling procedures of the DHS would ensure the interpretation with national representativeness. Finally, our multilevel analytical strategy allows us to vividly comprehend the effect each level exerts on sexual debut and teen birth, which help fill gaps in current literature mainly based on single-level studies (individual-level or ecological).

## Conclusions

We conclude that social norms are significantly associated with rapid sexual onset and teen birth. Further, adolescents residing in areas with more access to information and material resources (i.e., communities with higher proportion of inhabitants with secondary school or higher education) are also at high risk of early sexual debut. While social norms are embedded in the cultural context and therefore may require long-term interventions to be modified, the access to sexual education (oriented to increase awareness of the risks associated with early sexual debut) and supervision of quality connections to others outside the family may help to mitigate the erosion of traditional values observed in more developed communities [16]. Future studies using a richer set of social capital measures may be needed to validate these findings.
